# METTL3 depletion contributes to tumour progression and drug resistance via N6 methyladenosine-dependent mechanism in HR+HER2—breast cancer

**DOI:** 10.1186/s13058-022-01598-w

**Published:** 2023-02-10

**Authors:** Dengjie Ouyang, Tao Hong, Mengdie Fu, Yitong Li, Liyun Zeng, Qitong Chen, Hongye He, Ying Wen, Yan Cheng, Meirong Zhou, Qiongyan Zou, Wenjun Yi

**Affiliations:** 1 Department of General Surgery, The Second Xiangya Hospital, Central South University, No.139, Renmin Central Road, Changsha, 410011 China; 2 Department of General Surgery, Xiangya Hospital, Central South University, Changsha, China

**Keywords:** HR+HER2− breast cancer, m^6^A modification, METTL3, Drug sensitivity, EMT, Apoptosis

## Abstract

**Background:**

Chemotherapy is an important strategy for the treatment of hormone receptor-positive/human epidermal growth factor receptor 2-negative (HR+HER2−) breast cancer (BC), but this subtype has a low response rate to chemotherapy. Growing evidence indicates that *N*^6^-methyladenosine (m^6^A) is the most common RNA modification in eukaryotic cells and that methyltransferase-like 3 (METTL3) participates in tumour progression in several cancer types. Therefore, exploring the function of METTL3 in HR+HER2− BC initiation and development is still important.

**Methods:**

mRNA and protein expression levels were analysed by quantitative real-time polymerase chain reaction and western blotting, respectively. Cell proliferation was detected by CCK-8 and colony formation assays. Cell cycle progression was assessed by flow cytometry. Cell migration and invasion were analysed by wound healing assays and transwell assays, respectively, and apoptosis was analysed by TUNEL assays. Finally, m^6^A modification was analysed by methylated RNA immunoprecipitation.

**Results:**

Chemotherapy-induced downregulation of the m^6^A modification is regulated by METTL3 depletion in HR+HER2− BC. METTL3 knockdown in MCF-7/T47D cells decreased the drug sensitivity of HR+HER2− BC cells by promoting tumour proliferation and migration and inhibiting apoptosis. Mechanistically, CDKN1A is a downstream target of METTL3 that activates the AKT pathway and promotes epithelial-mesenchymal transformation (EMT). Moreover, a decrease in BAX expression was observed when m^6^A modification was inhibited with METTL3 knockdown, and apoptosis was inhibited by the reduction of caspase-3/-9/-8.

**Conclusion:**

METTL3 depletion promotes the proliferation and migration and decreases the drug sensitivity of HR+HER2− BC via regulation of the CDKN1A/EMT and m^6^A-BAX/caspase-9/-3/-8 signalling pathways, which suggests METTL3 played a tumour-suppressor role and it could be a potential biomarker for predicting the prognosis of patients with HR+HER2− BC.

**Supplementary Information:**

The online version contains supplementary material available at 10.1186/s13058-022-01598-w.

## Background

Globally, breast cancer (BC) is a highly heterogeneous disease with complex molecular bidirectional crosstalk between hormone receptors (HRs) and human epidermal growth factor receptor 2 (HER2) [1, 2]. Approximately 70% of patients have BC that is HR-positive and HER2-negative (HR+HER2−) [3]. Although endocrine-based therapies (aromatase inhibitors [AIs] and/or anti-oestrogen treatments with or without ovarian suppression) are the gold standard of treatment of BC and the backbone of adjuvant therapies for these patients with significantly decreased risk of recurrence and death [4], up to 20% of patients will eventually relapse [5, 6]. Single-agent chemotherapy is an essential treatment option for endocrine-resistant or treatment-refractory disease [7]. However, response rates to these therapies are low. Reported progression-free survival (PFS) ranges from 4.0 to 6.3 months with second-line chemotherapy and from 2.4 to 5.5 months with third-line chemotherapy [8]. Similarly, the association of pathological complete response (pCR) with disease-free survival (DFS) or overall survival (OS) in HR+HER2− BC following neoadjuvant systemic therapy is relatively low compared to that of the other two subtypes of BCs [9]. Thus, there is an imperative need to further improve our insight into the tumour biology and the tumour cell response to chemotherapy for HR+HER2− BC.

*N*^6^-Methyladenosine (m^6^A) is the most prevalent messenger RNA (mRNA) modification in eukaryotes and is added to mRNA molecules by the *N*^6^-adenosine methyltransferase complex, which consists of methyltransferase-like 3 (METTL3), methyltransferase-like 14 (METTL14) and Wilms tumour 1-associated protein (WTAP) [10]. METTL3 and METTL14 are two active methyltransferases that form a heterodimer to catalyse m^6^A RNA methylation, while WTAP interacts with this complex and substantially affects mRNA methylation [11]. Two m^6^A demethylases fat mass and obesity-associated (FTO) and AlkB homolog 5 (ALKBH5) have been discovered since 2011, revealing the dynamic nature of m^6^A modification [12]. Some cellular proteins have been found to preferentially bind m^6^A-modified RNA, whereas others have been characterized to specifically recognize m^6^A-modified mRNA and accelerate the decay of the mRNA [13]. These results indicated that this chemical modification is common and important in a variety of biological processes.

With the elucidation of the mechanisms involved in m^6^A modification, a recent report described the role of the m^6^A modification in multiple tumours [14]. Although studies on the function of m^6^A in BC are still in their early stages, there is growing evidence showing that m^6^A plays a critical role in many aspects of BC, including tumorigenesis [15], metastasis [16], prognosis [17] and treatment resistance [18]. Reduced expression of METTL3 promotes metastasis of BC by increasing COL3A1 expression [19]. Hypoxia stimulates ALKBH5, which stabilizes NANOG mRNA and induces a phenotype associated with BC stem cells (BCSCs) and lung metastasis [20, 21]. YTHDF3 promotes BC metastasis to the brain by inducing m^6^A-enriched gene translation [16]. Moreover, m^6^A modification patterns have therapeutic implications and correlate with drug resistance. HNRNPA2B1 and METTL3 overexpression in MCF-7 cells reduces their sensitivity to 4-hydroxytamoxifen and/or fulvestrant [22, 23]. In triple-negative BC (TNBC) cells, IGF2BP3 promotes chemoresistance to doxorubicin (DOX) and mitoxantrone by regulating ABCG2 expression [24]. Therefore, it is possible to determine why HR+HER2− BC is insensitive to chemotherapy treatment by further exploring the role of m^6^A modification in this subtype of BC.

In this study, we investigated the potential effect of m^6^A methylation on the tumour progression and sensitivity of HR+HER2− BC to chemotherapy. Our data revealed that chemotherapy decreased the levels of the m^6^A modification, which was dependent on METTL3 expression. We demonstrated that METTL3 depletion facilitates HR+HER2− BC progression via its downstream target cyclin-dependent inhibitor kinase 1A (CDKN1A), which mediates epithelial–mesenchymal transition (EMT). In addition, METTL3 reduction inhibits apoptosis by regulating BAX/caspase3/8/9 signalling in an m^6^A-independent manner. Overall, our results suggested that METTL3 plays a pivotal tumour-suppressor role in the progression of HR+HER2− BC, indicating that METTL3 is a promising biomarker for predicting the efficacy of chemotherapy as well as a potential therapeutic target for reversing chemotherapy resistance in HR+HER2− BC.

## Methods

### Human BC tissues

Thirteen pairs of primary BC tissues collected before and after treatment were obtained from the Second Xiangya Hospital of Central South University (Hunan, China) from August 2020 to March 2021. All individuals with BC were diagnosed for the first time, only received chemotherapy prior to surgery and had histologically confirmed HR+HER2− BC (Table1). All patients provided written informed consent, which was conducted in accordance with the Declaration of Helsinki, and this study was reviewed and approved by the Research Ethics Committee of the Second Xiangya Hospital of Central South University.

**Table1:** Clinicopathologic parameters of 13 HR+HER2− breast cancer patients

Variables	*n* (%)
Age (years)	
Median	52.00
Clinical T stage	
cT1-2	6 (46.2)
cT3-4	7 (53.8)
NAC regimen	
AC-T/P	11 (84.6)
TAC	2 (15.4)
NAC cycles	
≤ 4	9 (69.2)
> 4	4 (30.8)
Residual tumour size	
≤ 2 cm	1 (7.7)
> 2 cm	12 (92.3)
Nodal status	
Neg	5 (38.5)
Pos	8 (61.5)

*NAC* neoadjuvant chemotherapy

### Cell culture and chemicals

BC cell lines (MCF-7, T47D and MDA-MB-231) were obtained from the Shanghai Type Culture Collection of the Chinese Academy of Sciences and were grown in DMEM or RPMI 1640 medium (Gibco, Carlsbad, CA, USA) supplemented with 1% penicillin/streptomycin (Shanghai, Beijing, China). All cells in this study were incubated in 37 °C incubators with 5% carbon dioxide and routinely tested for mycoplasma. The chemotherapy drugs (doxorubicin [DOX], paclitaxel [PTX] and cisplatin [25]) were purchased from Dingguo (Beijing, China). The half-maximal inhibitory concentration (IC50) values of DOX were 1.0 and 0.2 μg/ml for MCF-7 and T47D cells, respectively. The IC50 values of PTX were 0.5 and 0.1 μM for MCF-7 and T47D cells, respectively, and the IC50 value of Cis was 400 nM for MCF-7 and T47D cells (Additional file 1A).

### Cell transduction

Stable knockdown and overexpression of METTL3 were achieved with lentiviral-based delivery of short-hairpin RNA (shRNA) and overexpression vectors, respectively. The shRNA sequences were subcloned into a lentiviral expression vector containing GFP by Shanghai Genechem Co., Ltd. (Shanghai, China). Lentiviral transduction was performed according to the manufacturer’s instructions. All constructed vectors were verified by DNA sequencing.

### Western blotting

Total proteins from cell lines and tissues were extracted with RIPA buffer and then quantified by BCA analysis. Subsequently, 20 µg of total protein per sample (10 µL per lane) was separated using sodium lauryl sulphate–polyacrylamide gel electrophoresis (10% polyacrylamide gel) before the proteins were transferred to a PVDF membrane. After incubation with primary antibodies overnight, the membranes were then incubated with secondary antibody. Finally, target protein bands were detected using a chemiluminescence system. The antibodies used targeted the following proteins: AKT (Cell Signaling #9272s), Caspase-9 (Proteintech 10380-1-AP), Caspase-3 (Proteintech 66470-2-Ig), Caspase-8 (Proteintech 66093-1-Ig), p-ATK (Cell Signaling #4060), BAX (Proteintech 60267-1-Ig), Vimentin (Proteintech 10366-1-AP), N-cadherin (Proteintech 22018-1-AP), E-cadherin (Proteintech 20874-1-AP), GAPDH (Signalway Antibody #21612) and METTL3 (ABclonal A8370).

### Quantitative real-time polymerase chain reaction (qRT-PCR)

Total RNA from cell lines and tissues was isolated using TRIzol reagent (Thermo Fisher Scientific, Beijing, China). A PrimeScript RT kit (Thermo Fisher Scientific, Beijing, China) was used for cDNA synthesis. Real-time quantitative PCR analysis was performed using a SYBR Premix Kit (Abclonal, Wuhan, China). Each sample was run in triplicate, and the expression levels were normalized to those of GAPDH using relative quantitative methods. All PCR primers (Well Biological Science, China) are listed as follows:

- GAPDH-F: ACAGCCTCAAGATCATCAGC
- GAPDH-R: GGTCATGAGTCCTTCCACGAT
- METTL3-F: TTGTCTCCAACCTTCCGTAGT
- METTL3-R: RCCAGATCAGAGAGGTGGTGTAG
- CDKN1A-F: CAAGCTCTACCTTCCCACGG
- CDKN1A-R: TCGACCCTGAGAGTCTCCAG
- BAX-F: ACTAAAGTGCCCGAGCTGA
- BAX-R: ACTCCAGCCACAAAGATGGT

### Cell proliferation assay

Cell proliferation and cytotoxicity assay kits (Dingguo, Beijing, China) were used to assess cell viability. Cells were seeded into 96-well plates at a density of 1 × 10^3^ cells per well and cultured in an incubator (37 °C with 5% CO_2_) for 24 h, 48 h and 72 h, after which cell proliferation was examined on a microplate reader by measuring the absorbance at a wavelength of 450 nm. To assess colony formation, cells were seeded into 6-well plates at a density of 1 × 10^3^ cells per well and cultured in an incubator (37 °C with 5% CO_2_). After 14 days, the cells were fixed and stained with Giemsa stain, modified solution (Sigma), and colonies containing 50 or more cells were counted.

### Cell cycle analysis

For cell cycle analysis, cells were seeded in 6-well plates at a density of 2–3 × 10^5^ per well and grown to ~ 70% confluence. Cells were then harvested and suspended in a complete medium. The cell suspension was centrifuged at 1300 rpm at 4 °C, washed once in D-Hanks buffer, counted and resuspended to a density of 3–6 × 10^6^ cells/mL. Ice-cold 70% ethanol was added dropwise to fix the cells, which were then centrifuged, washed once with D-Hanks, stained with 20 μg/mL propidium iodide (Sigma, P4170) in D-Hanks containing 50 μg/mL RNase A (Fermentas, EN0531) for 1 h at room temperature and analysed using flow cytometry.

### Transwell migration and invasion assay

For the tumour cell transwell migration and invasion assay, transfected breast cancer cells were seeded in DMEM/1640 without FBS in the upper chamber with or without a Matrigel coating. The lower chamber was filled with DMEM/1640 containing 10% FBS acting as a chemoattractant. After 24 h or 48 h, the cells in the upper chamber were washed away, and the remaining cells were fixed, dyed and photographed.

### Wound healing assay

For the wound healing assay, cells were seeded and cultured until a 90% confluent monolayer was formed. Cells were then scratched by a sterile pipette tip and treated as indicated in the text in an FBS-free medium. Cell migration distances into the scratched area were measured in 10 randomly chosen fields under a microscope.

### TUNEL assay

A TUNEL kit (Roche) was used according to the manufacturer’s instructions. In brief, cells were fixed with 4% paraformaldehyde, permeated with 0.1% Triton X-100 in PBS, incubated with 50 μL of TUNEL reaction mixture for 1 h at 37 °C and then washed with PBS 3 times. A total of 1000 cells were counted, and the percentage of apoptotic cells was quantified.

### In vivo tumour xenograft model

To assess the in vivo effects of METTL3, 4-week-old female BALB/c nude mice were used for tumour formation experiments. The experiments were carried out with the prior approval of the Second Xiangya Hospital of Central South University Committee on Animal Care, and the protocols were performed in accordance with the guidelines for the use of laboratory animals. For the experiments, mice were injected subcutaneously in the axilla with 1 × 10^7^ MCF-7 cells with stable expression of relevant plasmids. When the tumour diameter reached approximately 5 mm, nude mice were randomly divided into two groups (six mice per group). Xenografted mice were then administrated with PBS or DOX (5 mg/kg each mouse every 3 days) [26]. The health of the nude mice and the growth of the tumours were observed every 3 days for 21 days. Mice were killed after 3 weeks, and the tumours were separated, weighed and collected for experiment evaluation.

### Immunohistological staining

Fresh tumour tissues were excised from nude mice and fixed with 4% paraformaldehyde. Then, the sections were dehydrated with a graded alcohol series, cleared in xylene, embedded in paraffin and sliced into sections with a microtome at a thickness of approximately 4 µm. After more treatments with xylene and an alcohol gradient, the sections were dewaxed and hydrated. Ki-67 antibody was added and then incubated overnight, and the secondary antibody was incubated for 1 h. Diaminobenzidine (DAB) was used for colour development, and the slides were stained with haematoxylin before they were mounted in neutral resin. With a common microscope, 40× high-definition fields were randomly selected for counting the total number of cells and the number of Ki-67-positive cells (which have brown nuclei).

### RNA m^6^A quantification, methylated RNA immunoprecipitation (MeRIP) and quantitative PCR

The m^6^A content of 200 ng of RNA extracted from tissues and cell lines was measured by an EpiQuik m^6^A RNA methylation quantification kit (EpigenTek, USA) according to the manufacturer’s instructions. Immunoprecipitation of m^6^A-modified BAX and CDKN1A mRNA was performed using a Tiangen MeRIP m^6^A Kit (FP313, Tiangen, China) according to the manufacturer’s protocol. m^6^A enrichment was analysed by qPCR with specific primers (Well Biological Science, China), and data were normalized to the input. Primer sequences were as follows:

- BAX-Positive-F: AGGATCGAGCAGGGCGAAT
- BAX-Positive-R: AGCTGCCACTCGGAAAAAGA
- BAX-Negative-F: CCGAGTCACTGAAGCGACTG
- BAX-Negative-R: ACGTGGGCGTCCCAAAGTAG
- CDKN1A-Positive-F: TCTTCGGCCCAGTGGACA
- CDKN1A-Positive-R: AGTCGAAGTTCCATCGCTCA
- CDKN1A-Negative-F: TCCTCATCCCGTGTTCTCCT
- CDKN1A-Negative-R: ACAAGTGGGGAGGAGGAAGT

### Bioinformatics analysis

The expression profiles of m^6^A-related mRNAs were obtained from the GSE87455 and GSE763 data sets (https://www.ncbi.nlm.nih.gov/geo/), which comprised 69 pairs of pre- and post-chemotherapy BC tissues and MCF-7 cells treated with DOX, respectively. Differential expressions of m^6^A-related genes between the pre-treatment and post-treatment tissues was determined by R software (http://www.r-project.org/). The association of METTL3 expression with patient OS and RFS was analysed with Kaplan–Meier Plotter (http://kmplot.com/analysis/index.php?p=service&cancer=pancancer_rnaseq) based on the TCGA data. Gene Ontology (GO) analysis and Kyoto Encyclopedia of Genes and Genomes (KEGG) enrichment analysis of differentially expressed genes (DEGs) were performed using the DAVID database (https://david.ncifcrf.gov/).

### Statistical analysis

Experimental data are presented as the mean ± standard deviation (SD) and were analysed using GraphPad Prism 8.0 software. All in vitro results are representative of at least three independent trials. Two-group comparisons were assessed by the Mann–Whitney U test or Student’s t test, and paired t tests were employed for paired BC and corresponding chemo-only BC samples. A two-tailed *p* value of 0.05 was considered statistically significant.

## Results

### Chemotherapy-induced decreases in METTL3 expression correlated with a poor prognosis in HR+HER2− BC

**Fig. 1:**
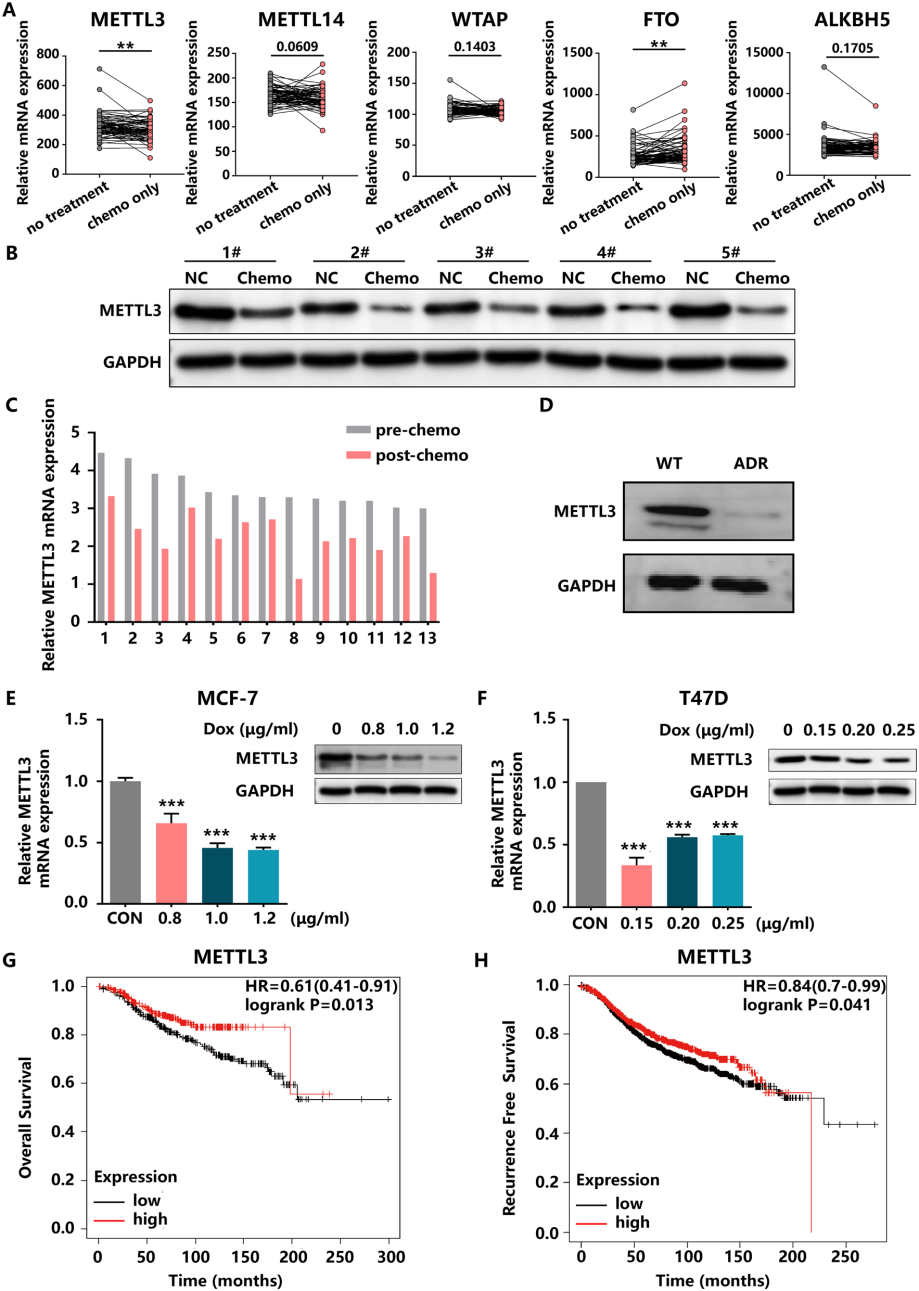
METTL3 is silenced in HR+HER2− BC samples and cell lines. **A** The expression of m^6^A-related genes in the GSE87455 BC cohort. **B** and **C** The protein and mRNA levels of METTL3 in paired HR+HER2− BC tissues obtained before and after chemotherapy treatment. **D** The protein level of METTL3 in MCF-7 and MCF-7/ADR cell lines. **E** and **F** The mRNA and protein levels of METTL3 in MCF-7 and T47D cells treated with different concentrations DOX for 24 h and their corresponding control cells. **G** and **H** Upregulation of METTL3 expression was significantly associated with longer OS and RFS in HR+HER2− BC patients

To identify the crucial regulator of m^6^A modification in the progression of HR+HER2− BC, we screened the expression profiles of m^6^A writers (METTL3, METTL14 and WTAP) and m^6^A erasers (FTO and ALKBH5) in the GSE87455 platform. The results of the analysis showed that METTL3 was significantly reduced and FTO was increased in the chemo-only group, whereas METTL14, WTAP and ALKBH5 expression levels showed no significant differences between the untreated specimens and chemo-only tumour specimens (Fig. 1A). We also examined these m^6^A-related genes in the GSE763 data set (Additional file 1B), and only the pattern of METTL3 expression was consistent with that in the GSE87455 cohort. Next, we detected METTL3 expression in 13 pairs of pre- and post-chemotherapy HR+HER2− BC tumour tissues and observed that METTL3 expression was deficient in the post-chemotherapy tissues compared to the pre-treatment tissues (Fig. 1B, C Additional file 1C). In addition, the downregulation of METTL3 in tissues after chemotherapy was consistent with the expression pattern in the MCF-7/ADR cell line (Fig. 1D). MCF-7 and T47D cell lines treated with chemotherapeutic drugs, including DOX (Fig. 1E, F), PTX (Additional file 1D) and Cis (Additional file 1E), corroborated these findings. qRT-PCR and western blotting confirmed that METTL3 expression was reduced at the mRNA and protein levels in the drug-treated group compared with the control group. Furthermore, individuals with HR+HER2− BC and low METTL3 expression had a worse prognosis, and METTL3 expression was correlated with recurrence-free survival (RFS) (Fig. 1G, H).

### METTL3 depletion attenuated the drug sensitivity of HR+/HER2− BC cells by promoting tumour proliferation, migration, invasion and inhibiting apoptosis

**Fig. 2:**
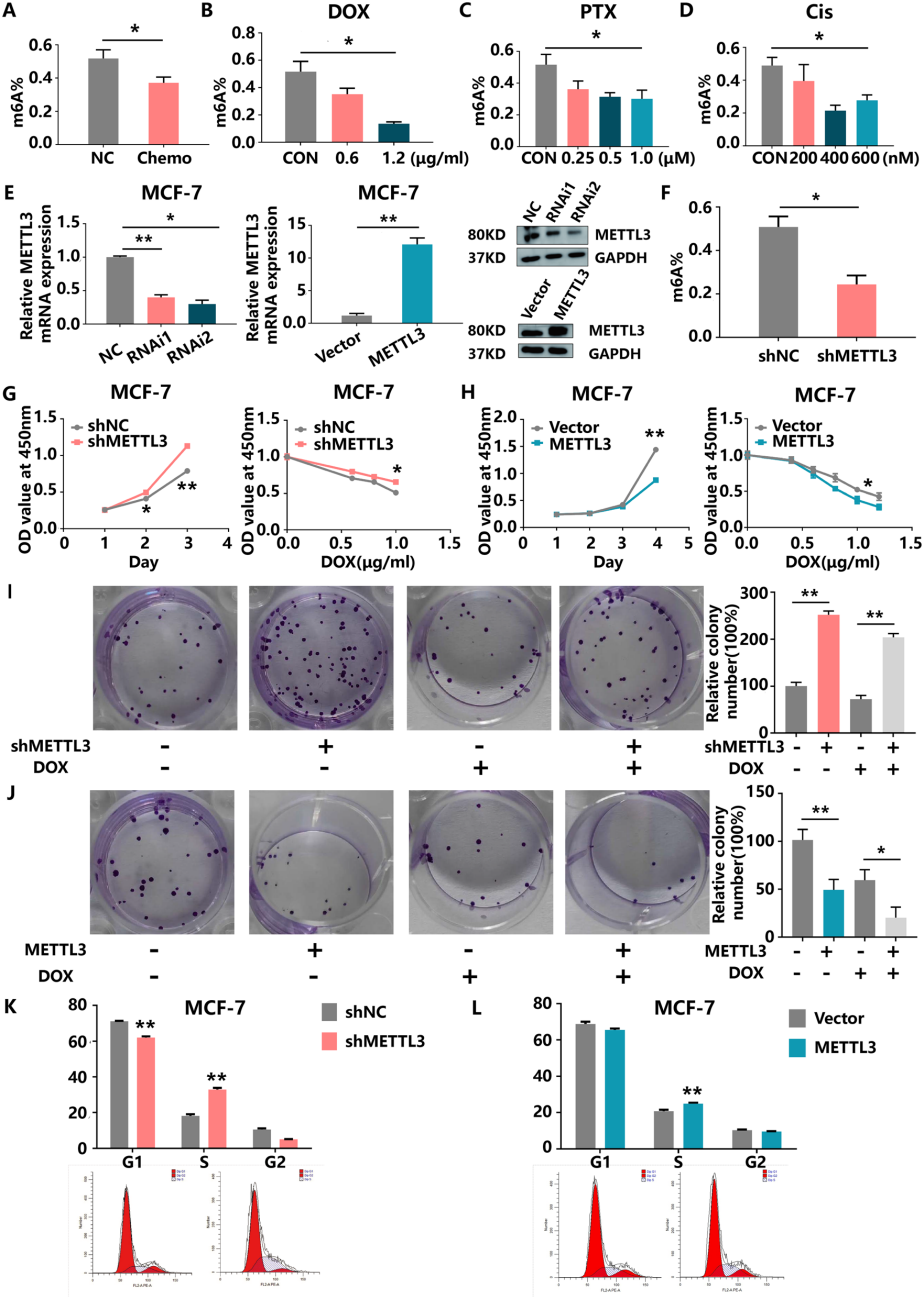
METTL3 is responsible for chemotherapy-induced m^6^A modifications, and METTL3 depletion enhances HR+HER2− BC cell growth. **A** m^6^A levels in patient samples collected after chemotherapy compared with their corresponding controls. **B C** and **D** m^6^A levels in MCF-7 cells treated with DOX, PTX or Cis for 24 h compared with those in the control cells. **E** The protein and mRNA levels of METTL3 in MCF-7 cells with knockdown or overexpression of METTL3 were measured by western blotting and qRT-PCR, respectively. **F** The global m^6^A modification levels in METTL3-knockdown and control MCF-7 cells were determined by colorimetric analysis. **G** Knockdown of METTL3 improved the proliferation ability of MCF-7 cells in the presence or absence of DOX for 24 h. **H** Overexpression of METTL3 impaired the proliferation ability of MCF-7 cells in the presence or absence of DOX for 24 h. **I** Knockdown of METTL3 improved the colony formation ability of MCF-7 cells in the presence or absence of DOX for 24 h (left panel). Quantification of the colony formation assay results (right panel). **J** Overexpression of METTL3 impaired the colony formation ability of MCF-7 cells in the presence or absence of DOX for 24 h (left panel). Quantification of the colony formation assay results (right panel). **K** and **L** Cell cycle distribution of MCF-7 cells with knockdown or overexpression of METTL3 was analysed by flow cytometry. Ap, apoptosis phase; G1, DNA pre-synthesis phase; S, DNA synthesis phase; G2, DNA post-synthesis phase

We first detected the global m^6^A level in BC tissues and cell lines using an m^6^A RNA methylation quantification kit. The levels of m^6^A-modified RNA were remarkably higher in untreated BC tissues than in matched chemo-only tumour tissues (Fig. 2A) and in control MCF-7 cells than in cells treated with chemotherapeutic drugs (Fig. 2B–D). Similar results were observed in T47D cells treated with DOX, PTX and Cis (Additional file 2A). However, decreased global m^6^A levels were not observed in MDA-MB-231 cells, which are a TNBC cell line (Additional file 2B). To clarify the role of METTL3 in HR+HER2− BC tumorigenesis, we inhibited and upregulated METTL3 expression via transfection of shMETTL3 and LV-METTL3 vectors, respectively, in MCF-7 and T47D cells. The knockdown and overexpression efficiencies at the mRNA and protein levels were determined by qRT-PCR and western blot in these two cell lines (Fig. 2E, Additional file 2C). The levels of m^6^A modification were decreased upon METTL3 inhibition (Fig. 2F). Notably, METTL3 knockdown obviously enhanced cell proliferation but reduced the sensitivity of MCF-7 cells to DOX as shown in the CCK-8 assays (Fig. 2G). As anticipated, METTL3 knockdown enhanced colony formation, especially in the DOX-treated group (Fig. 2I), whereas METTL3 overexpression evoked the opposite effects (Fig. 2H, J). Furthermore, a cell cycle assay demonstrated that silencing METTL3 prominently increased the proportion of cells in the S phase (Fig. 2K). However, METTL3 upregulation did not obviously reduce the proportion of S-phase cells (Fig. 2L). Similarly, we also observed a protective effect of METTL3 in T47D cells (Additional file 2D–I).

**Fig. 3:**
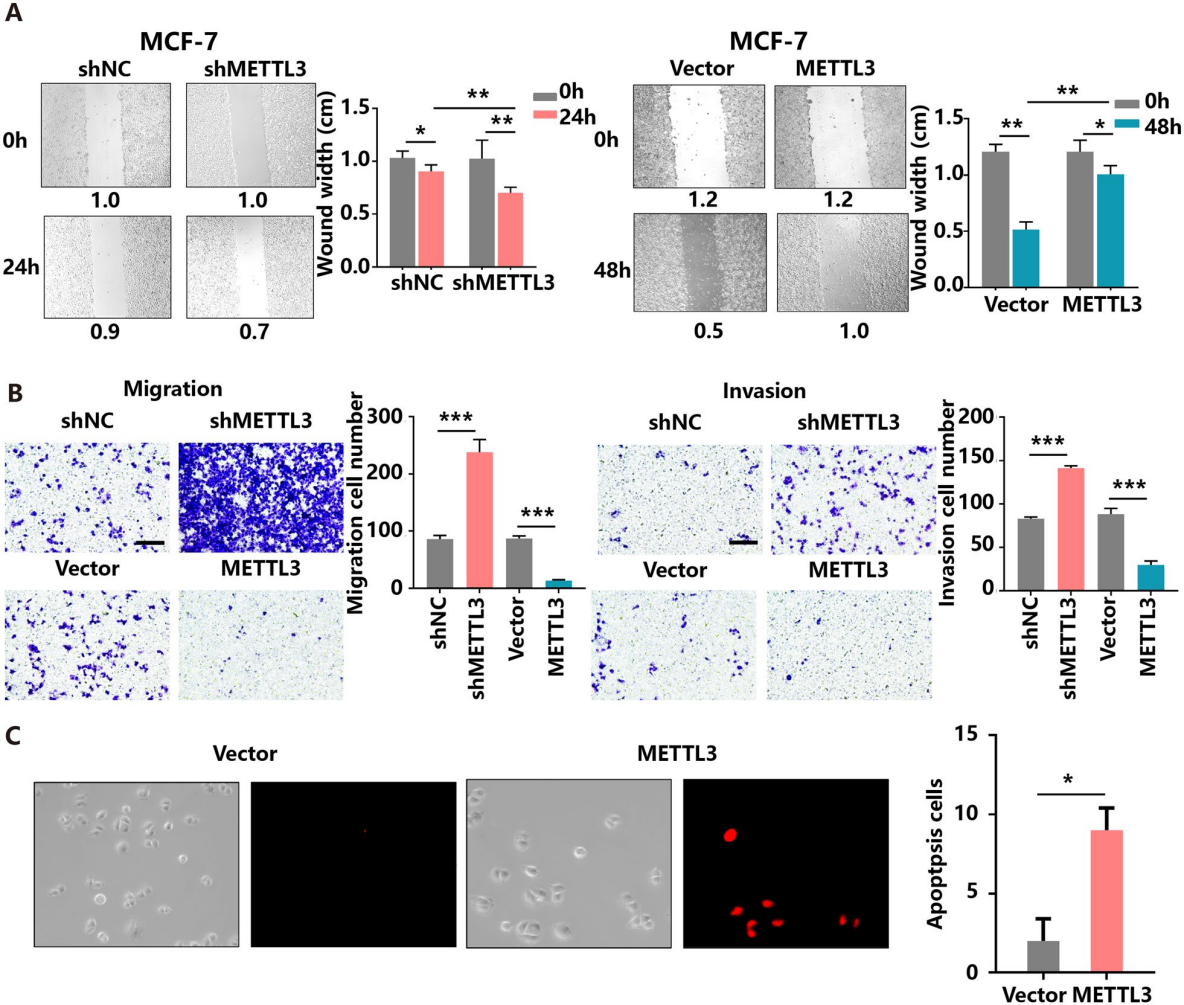
Inhibition of METTL3 drives BC cell migration and invasion in vitro. **A** Knockdown or overexpression of METTL3 affects MCF-7 cell migration in vitro as indicated by the wound healing assay. **B** Knockdown or overexpression of METTL3 affects MCF-7 cell migration and invasion in vitro as indicated by the Transwell assay. **C** Overexpression of METTL3 promotes MCF-7 cell apoptosis

A major factor shaping the malignancy character of cancer cells lies in their motility. Changes in the gene expression and signalling pathways directing its regulation can lead to the pathological processes of tumour cell invasion and migration [27]. Wound healing and Transwell assays were utilized to assess the effects of METTL3 on BC cell mobility. The results demonstrated that depletion of METTL3 induced, but overexpression of METTL3 inhibited, MCF-7 and T47D cell migration and invasion in vitro (Fig. 3A, B Additional file 3A–B). Moreover, an increase in the number of apoptotic cells was observed among METTL3-overexpressing MCF-7 and T47D cells (Fig. 3C, Additional file 3C). Taken together, these findings suggest the anti-proliferative and anti-migratory roles of METTL3 in BC cells.

### The increment of METTL3 expression inhibits tumour growth and enhances sensitivity to DOX in vivo

**Fig. 4:**
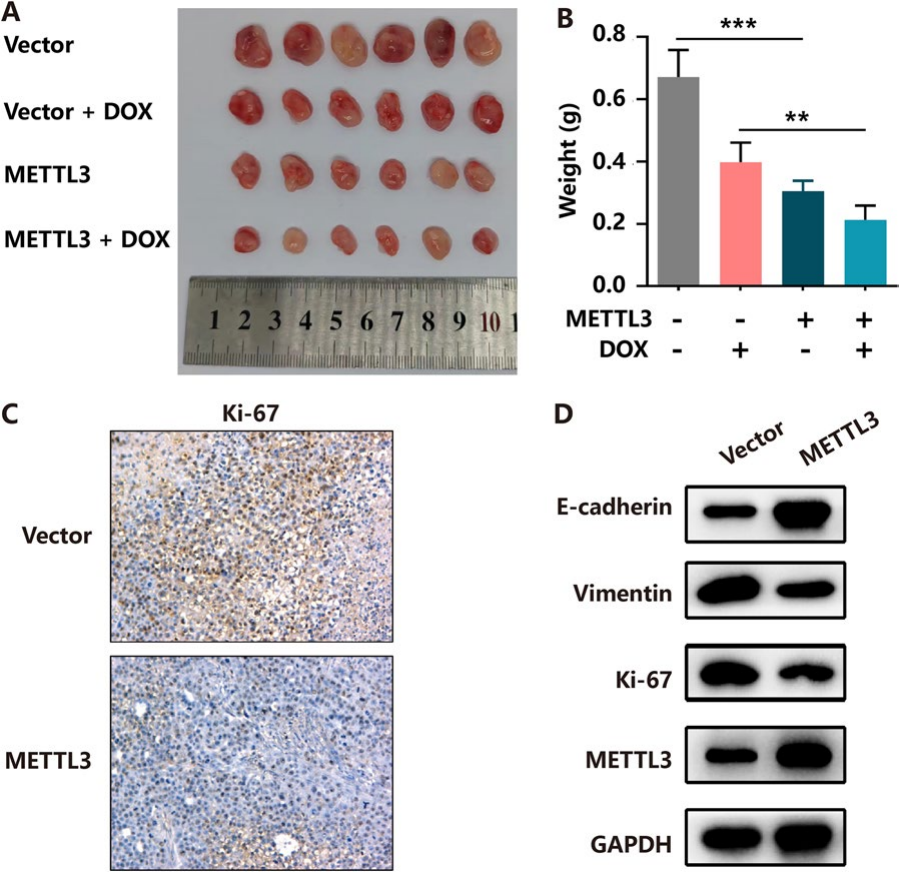
Increasing of METTL3 inhibits BC cell growth in vivo. **A** Overexpression of METTL3 effectively inhibited HR+HER2− BC subcutaneous tumour growth and increased sensitivity to DOX in nude mice (*n* = 6). **B** The tumour weight was measured in the NC and METTL3 groups with or without DOX-treated. **C** and **D** Tumours were excised and processed for immunohistochemical staining and western blot for Ki-67, Vimentin and E-cadherin

Based on the aforementioned in vitro findings, METTL3 was suggested to inhibit breast cancer cell progression and enhance the susceptibility to DOX treatment. To further confirm the relationship among METTL3, tumour growth and DOX sensitivity in human breast cancer, we generated a mouse xenograft model injected by MCF-7 cell line with the stable expression of METTL3 or a control vector. Approximately a week after the subcutaneous implantation of these cells into mice, slower tumour growth and smaller tumours were observed in the METTL3 group as compared to vector group. To further explore whether METTL3 decreases resistance to DOX in breast cancer, we treated mice with PBS and DOX. The smallest tumours were observed in the METTL3 group treated with DOX (Fig. 4A, B). Additionally, semi-quantitative IHC analysis and WB assays of Ki-67, vimentin expression levels in the xenografts revealed that METTL3 group exhibited lower levels of these proteins than in the vector group, with opposite results observed for E-cadherin (Fig. 4C, D). Taken together, these results indicate that upregulation of METTL3 combined with DOX significantly suppresses the tumour growth.

### Multidimensional sequencing identifies CDKN1A as a downstream target of METTL3

**Fig. 5:**
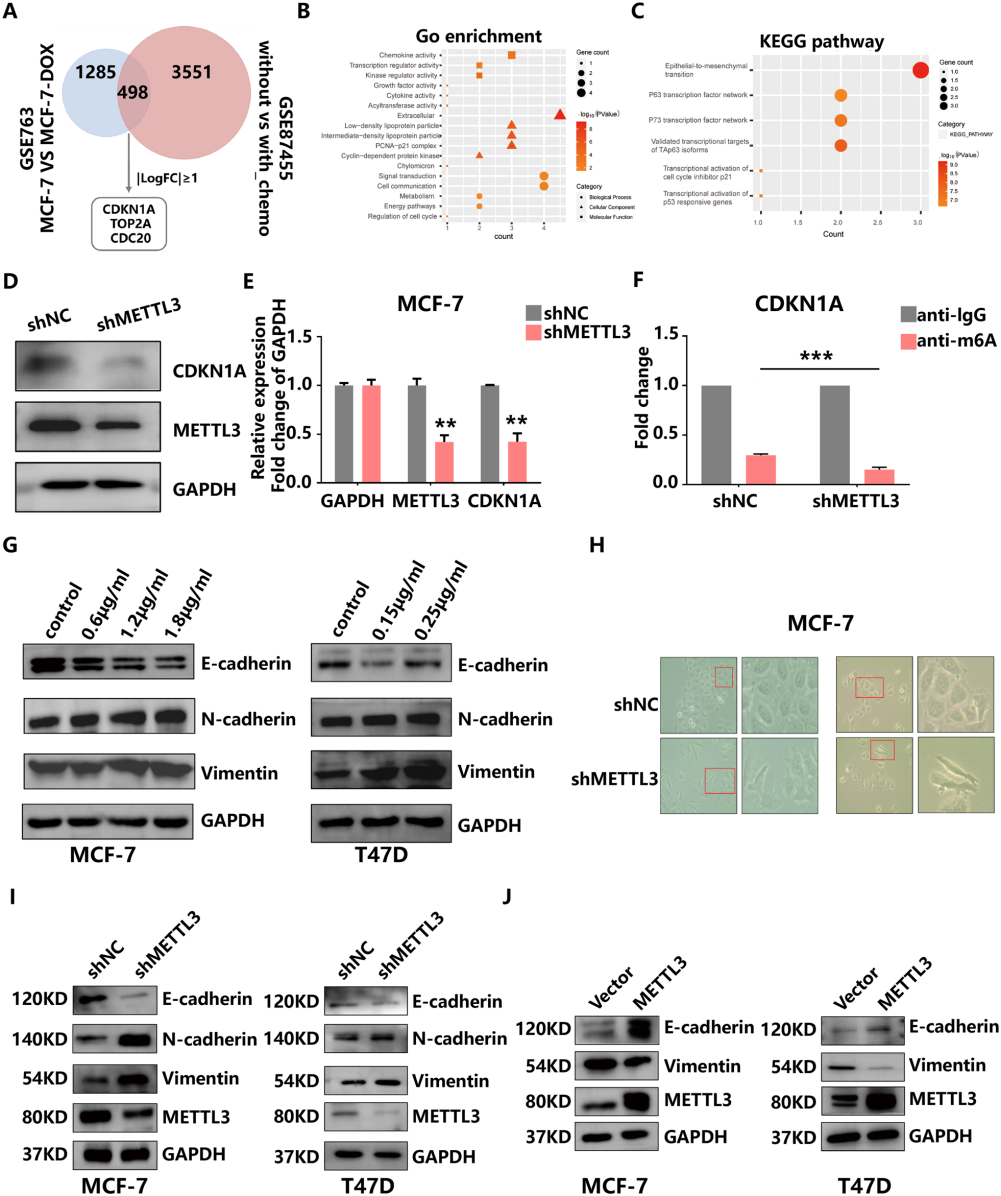
Identification of potential targets of the METTL3-mediated m^6^A modification in BC. **A** Venn diagram showing 498 overlapping genes with differential expression between the GSE763 and GSE87455. **B** and **C** GO and KEGG analysis of the overlapping genes. **D** and **E** The protein and mRNA levels of METTL3 and CDKN1A in MCF-7 cells with METTL3 knockdown were measured by western blot and qRT-PCR, respectively. **F** MeRIP with an anti-m^6^A antibody was performed in MCF-7 cells. The m^6^A modification on CDKN1A mRNA was reduced upon METTL3 knockdown. **G** E-cadherin, N-cadherin, Vimentin and GAPDH protein levels were examined by western blot in DOX-treated and control MCF-7 and T47D cell lines after 24 h. **H** Cell morphologic changes after downregulation of METTL3. **I** E-cadherin, N-cadherin, Vimentin, METTL3 and GAPDH protein levels were examined by western blot in NC and knockdown METTL3 groups of HR+HER2− BC cells. **J** E-cadherin, Vimentin, METTL3 and GAPDH protein levels in the vector and overexpression METTL3 groups were examined by western blotting

To better understand the possible mechanism by which METTL3 participates in HR+HER2− BC progression, we first performed DEG analysis of the GSE87455 and GSE763 data sets and identified 4049 and 1783 DEGs in these two cohorts, respectively. There were 498 overlapping genes altered by chemotherapeutic drugs in both data sets, among which cyclin-dependent kinase inhibitor 1A (CDKN1A), DNA topoisomerase II alpha (TOP2A) and cell division cycle 20 (CDC20) were filtered by the criteria |log_2_FC|≥ 1 and *p* value < 0.05 (Fig. 5A). GO and KEGG enrichment analysis revealed that the DEGs were mostly linked to EMT, transcriptional activation of cell cycle inhibitor p21 and transcriptional activation of p53 responsive genes, indicating a regulatory role of METTL3 in the progression of HR+HER2− BC (Fig. 5B, C). Because CDKN1A exhibited the most distinct differential expression, we chose this gene as a candidate target for subsequent experiments. RT-PCR and western blot experiments confirmed the positive regulation of METTL3 on CDKN1A at the mRNA and protein levels (Fig. 5D, E). Additionally, MeRIP-qPCR using a m^6^A-specific antibody showed that the levels of m^6^A-modified CDKN1A were decreased in METTL3-silenced MCF-7 cells (Fig. 5F). These data suggest that METTL3 regulates CDKN1A mRNA expression via m^6^A methyltransferase activity.

EMT plays a critical role in the tumour progression and drug resistance [28]. We also found that DOX induced a decrease in the expression of E-cadherin and an increase in the expression of N-cadherin and vimentin in MCF-7 and T47D cells (Fig. 5G) indicative of EMT. Similarly, inhibition of METTL3 resulted in increased protein levels of N-cadherin and vimentin, whereas overexpression of METTL3 showed the opposite trend (Fig. 5I, J). Furthermore, pseudopodia and cell spacing in MCF-7 cells were increased in response to METTL3 knockdown (Fig. 5H). These dynamic biochemical and morphological changes enable breast tumour cells to assume a mesenchymal phenotype with enhanced migratory and invasive capabilities [27], which were confirmed in the above experiments. Collectively, these results suggested the importance of METTL3-mediated EMT signalling in chemoresistance.

### METTL3 regulates EMT in a PI3K/AKT signalling-dependent manner

**Fig. 6:**
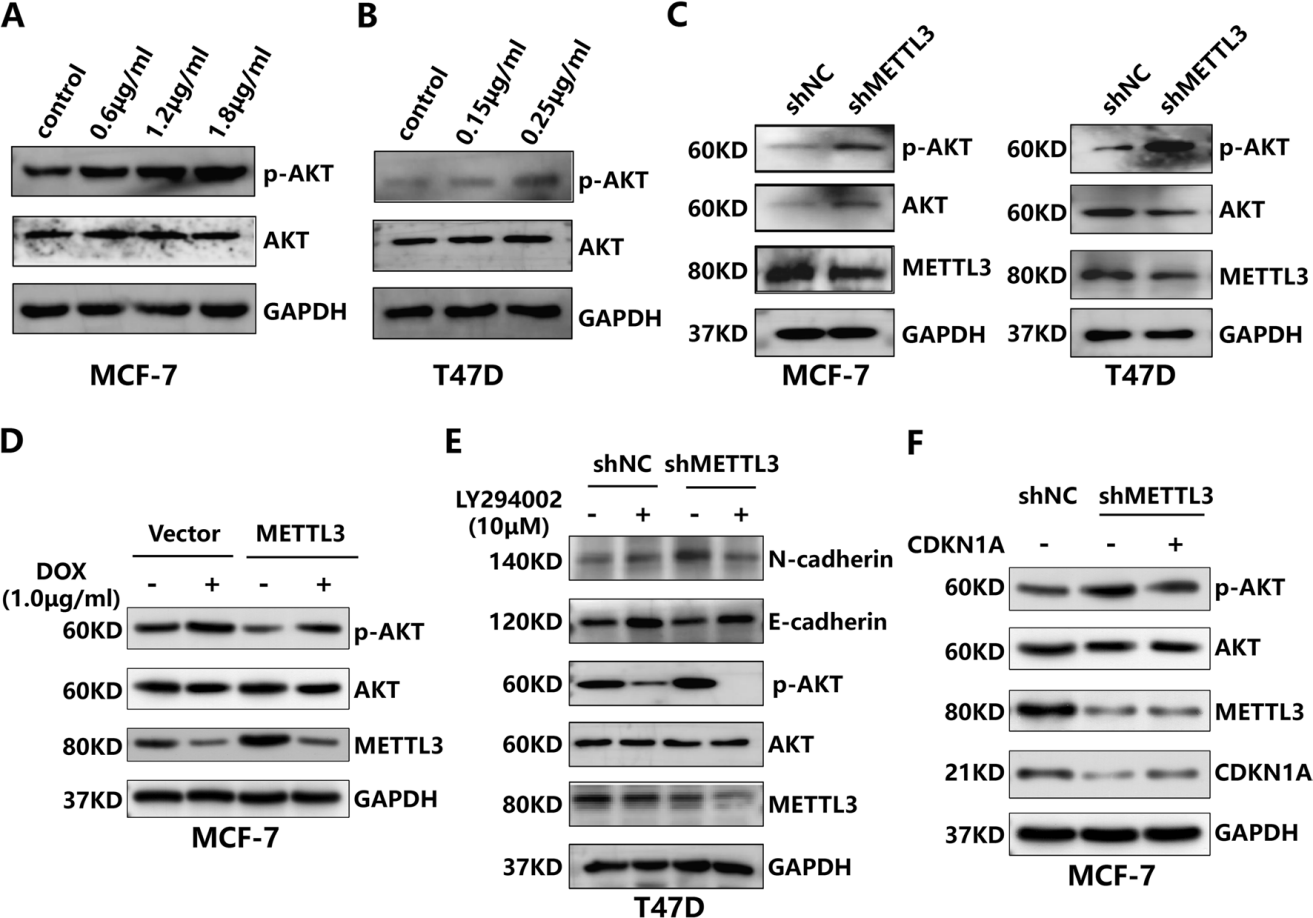
DOX influences AKT activation and promotes EMT progression in HR+HER2− BC cells. **A** and **B** p-AKT, AKT and GAPDH protein levels were examined by western blot in DOX-treated and control HR+HER2− BC cells after 24 h. **C** p-AKT, AKT, METTL3 and GAPDH protein levels were examined by western blot in in NC and knockdown METTL3 groups of HR+/HER2− BC cells. **D** p-AKT, AKT, METTL3 and GAPDH protein levels in the NC, NC-DOX, METTL3 overexpression and METTL3 overexpression -DOX groups were examined by western blotting. **E** E-cadherin, N-cadherin, p-AKT, AKT, METTL3 and GAPDH protein levels in the shNC, shNC-LY294002, shMETTL3 and shMETTL3-LY294002 groups were examined by western blotting. **F** p-AKT, AKT, METTL3, CDKN1A and GAPDH protein levels in the NC, shMETTL3 and shMETTL3-CDKN1A groups were examined by western blotting

Despite the observed critical role of METTL3 in the EMT process, whether this activity is specifically attributed to the METTL3/CDKN1A axis needs to be further explored. HR+HER2− BC is insensitive to chemotherapy, which may be largely related to the mechanism of cellular feedback regulation to drug exposure. There is often a mutation in the PIK3CA gene in HR+HER2− BC, which plays a crucial role in regulating the PI3K/AKT pathway and drug resistance [29]. However, the precise role of DOX-induced PI3K/AKT pathway activity has not been documented. Therefore, we first determined the effect of DOX intervention on the levels of p-AKT and total AKT. We observed increased levels of p-AKT in MCF-7 and T47D cells treated with DOX, as evidenced by the western blot experiments (Fig. 6A, B). We also assessed the potential effects of METTL3 on the PI3K/AKT pathway. The data showed that METTL3 depletion resulted in upregulation of p-AKT levels (Fig. 6C). Conversely, MCF-7 cells with stable overexpression of METTL3 showed restrained p-AKT levels compared with those of the other groups (Fig. 6D). Subsequently, we tested the influence of METTL3 and PI3K/AKT on the EMT pathway using the PI3K inhibitor LY294002. The data showed that the increased N-cadherin and p-AKT levels associated with METTL3 deficiency could be partially attenuated by treatment with LY294002 (Fig. 6E), highlighting the essential role of PI3K/AKT in the METTL3-controlled EMT process. Additionally, we conducted bibliometric analysis and found that CDKN1A is one of the key molecules in the PI3K/AKT pathway (Additional file 4A). Genetic upregulation of CDKN1A also impaired the effects of METTL3 inhibition on p-AKT activation (Fig. 6F). Our results clarify the critical role of CDKN1A and AKT in METTL3-mediated EMT progression in HR+HER2− BC.

### BAX activates caspase3/8/9 to facilitate METTL3-mediated tumour cell apoptosis

**Fig. 7:**
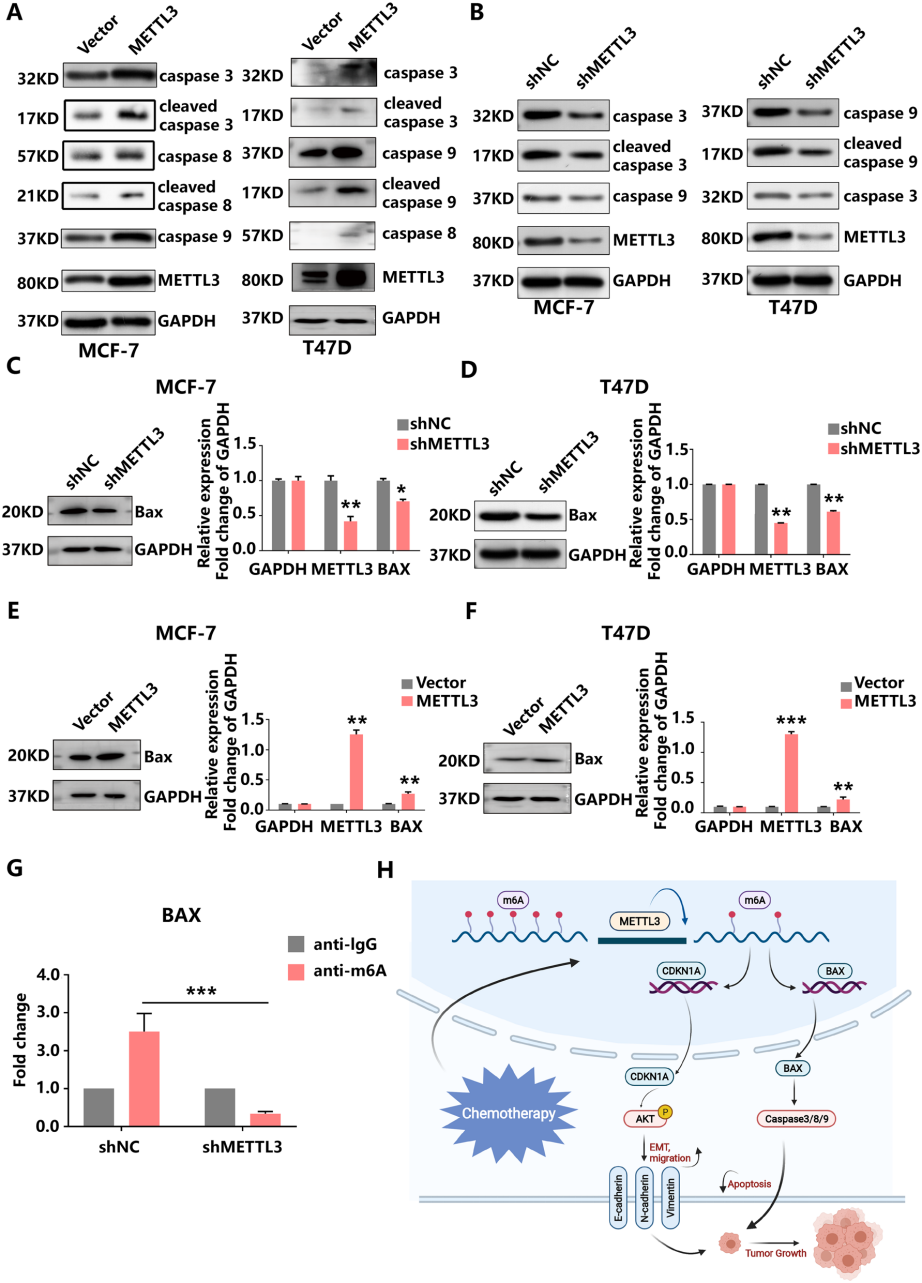
METTL3 induces HR+HER2− BC apoptosis via the m^6^A-Bax/caspase-3/-8/-9 pathway. **A** and **B** METTL3 knockdown or overexpression influenced the expression levels of caspase proteins as indicated by the western blot assay. **C** and **D** BAX expression was depleted upon METTL3 knockdown but enhanced by METTL3 overexpression. **E** MeRIP with an anti-m^6^A antibody was performed in MCF-7 cells. The m^6^A modification on BAX mRNA was depleted upon METTL3 knockdown. **F** Schematic summarizing the role of METTL3 in modulating HR+HER2− BC progression

We demonstrated that METTL3 promoted apoptosis (Fig. 3C, Additional file 3C). Given the involvement of the apoptosis signalling pathway in tumour proliferation, western blotting was implemented to visualize changes in the expression of apoptosis-related proteins following METTL3 knockdown or overexpression in MCF-7 and T47D cells. We found that increasing METTL3 expression elevated the levels of multiple apoptosis-related proteins, including caspase-3, caspase-8 and caspase-9. By contrast, the knockdown of METTL3 appreciably decreased the expression of these proteins (Fig. 7A, B). BAX is an important protein that promotes apoptosis. As an upstream gene of caspase3, BAX regulates the activation of the caspase family. To further verify the relationship between METTL3 and apoptosis, the expression of apoptosis-related genes was detected at the mRNA and protein levels. We observed that increased METTL3 expression could facilitate the expression of BAX in MCF-7 cells and T47D cells (Fig. 7C–F). Notably, MeRIP-qPCR confirmed the interaction between BAX mRNA and m^6^A in METTL3-depleted MCF-7 cell lines (Fig. 7G). Therefore, we demonstrate that METTL3 increases BAX/caspase3/8/9 expression, promotes apoptosis and restrains tumorigenesis in HR+HER2− BC cells.

## Discussion

In recent decades, substantial improvements have been made in therapeutic interventions for BC, which have increased the survival and quality of life of patients [30]. Chemotherapy, as the broadest application of tumour treatment, remains the cornerstone of adjuvant therapy for BC and is widely used in BC patients with high metastatic burden and locally advanced disease [31]. Nevertheless, recent studies have indicated that the response rate of BC patients with the HR+HER2− subtype to chemotherapy is low [32]. Hence, the demand for elucidating the mechanism of HR+HER2− BC insensitivity to chemotherapy is crucial. In this study, we first discovered that m^6^A modifications and METTL3 expression were inhibited by chemotherapy; thus, we evaluated the function of METTL3 in regulating HR+HER2− BC progression and drug sensitivity. Based on our findings, we revealed that METTL3 plays a protective role in HR+HER2− BC and regulates the CDKN1A/EMT and BAX/caspase3/8/9 axes in an m^6^A-dependent manner, which could be novel pathways involved in a potential mechanism of HR+HER2− BC chemoresistance (Fig. 6H).

m^6^A modification, one type of RNA epigenetic modification, has been identified on almost all types of RNAs and has been implicated in a variety of cellular processes, including mRNA stability, splicing, location, and translation, RNA–protein interactions and pri-miRNA processes (28–33). An increasing number of studies have addressed the pathological significance of m^6^A dysregulation in human diseases, especially in cancers [33–35]. The results of our current study showed that the overall level of m6A modification was significantly downregulated after chemotherapy in HR+/HER2− BC patients, and treatment of MCF-7 and T47D cells with DOX, PTX and Cis also resulted in a decrease in m^6^A modifications. However, the levels of m^6^A modification were not affected by drug intervention in MDA-MB-231 cells. Therefore, our results suggested that chemotherapy-induced changes in m^6^A levels are a biological difference between HR+HER2− BC and TNBC, especially in terms of responsiveness to chemotherapy. Various studies indicate that the m^6^A modification affects drug sensitivity by regulating ABC transporters either directly at the transcript level or via upstream signalling pathways [36]. Recent studies also indicated that the m^6^A modification is involved in the maintenance of CSCs in tumours, leading to drug resistance and recurrence [37]. It has also been shown that m^6^A modifications can affect the response of BC to endocrine therapy [22]. However, there are few studies on the relationship between m^6^A and chemotherapy response. Therefore, considering the potential role of the m^6^A RNA modification in the development of chemoresistance, it is necessary to illustrate the relationship between these two phenomena.

METTL3, a key component of the *N*^6^-methyltransferase complex, has been reported to play an important role in many tumour types [38–43]. Previous studies reported that METTL3 plays an oncogenic role in acute myeloid leukaemia through diverse downstream targets [43], whereas other studies suggested that either increased or decreased METTL3 expression could promote the self-renewal and tumorigenicity of glioma stem-like cells, respectively [42, 44]. Regarding METTL3 in BC, data from the literature have suggested that METTL3 can promote BC progression by targeting Bcl-2, HBXIP or SOX2 [41, 45, 46] and that METTL3 could promote adriamycin resistance by accelerating pri-miRNA-221-3p maturation [47] or mediating MALAT1/E2F1/AGR2 axis [48]. However, our results illustrated that chemotherapy-mediated depletion of METTL3 plays a significant unprotective role in tumour progression and drug tolerance. Reasonable explanations for these contradictory phenomena could be attributed to recognition by different m^6^A readers [49]. We speculated that the m^6^A modification and METTL3 expression protect some critical genes from degradation or restrain the role of oncogenes by enhancing their recognition by “readers”. We analysed the differential expression of “readers” in two data sets (GSE87455 and GSE763), and the results showed that only HNRNPA2B1 expression was significantly decreased after chemotherapy in both data sets. We also verified the expression of HNRNPA2B1 in cell lines after DOX intervention (Additional file 4B, C). Current studies have shown that HNRNPA2B1 participates in gene processing and alternative splicing and is a negative regulator of human breast cancer metastasis by maintaining the balance of multiple genes and pathways [50, 51], and the relationship between HNRNPA2B1 and chemotherapy in breast cancer is not clear. Therefore, we speculated that the downregulation of HNRNPA2B1 caused the dysregulation of CDKN1A and BAX, but the specific mechanism still needs further verification. In summary, the decreased METTL3 expression is secondary to chemotherapy, which is consistent with the clinical medication pattern, and HR+HER2− BC is the only BC subtype to exhibit this expression pattern. Therefore, METTL3 can be used as a biomarker to predict the sensitivity of HR+HER2− BC to chemotherapy and as a novel target for combination therapy to reverse chemotherapy resistance.

Our results further showed that METTL3 regulates the proliferation, apoptosis, migration and drug sensitivity of HR+/HER2− BC through multiple signalling pathways. On the one hand, METTL3 can affect the m^6^A modification of BAX mRNA, thereby promoting activation of the pro-apoptotic caspase cascade and (consequently) apoptosis. Apoptosis is an important mechanism to mitigate the uncontrolled growth of tumour cells and is mainly regulated by the Bcl2 protein family [52]. The Bcl2 protein family can be divided into two categories according to their functions: one plays a pro-apoptotic role and includes BAX and Bak, whereas the other plays an anti-apoptotic role and includes Bcl2. Both pathways promote caspase cascades that eventually lead to cell death [52]. Our experiment found that METTL3 can promote the expression of BAX and the subsequent activation of caspase3, 8, and 9, leading to apoptosis.

On the other hand, downregulation of METTL3 can regulate CDKN1A expression to affect the EMT process and promote cell proliferation, migration and invasion. CDKN1A is one of the key molecules involved in cell cycle progression and was first identified as a tumour suppressor [53]. Later, it was found to be involved in pathways related to tumorigenesis and development, such as cell death, DNA replication/repair, gene transcription and cell motility [54]. It is believed that the role of CDKN1A depends on its cellular localization [55]. When in the nucleus, CDKN1A functions as a tumour suppressor. However, when CDKN1A is concentrated in the cytoplasm, p53-impaired or p53-deficient cells may acquire carcinogenic properties, which may inhibit apoptosis and promote cell migration and proliferation [56]. Studies have shown that miR-33b-3p can promote the survival and cisplatin resistance of A549 human lung cancer cells by targeting CDKN1A after DNA damage [57]. It was also found that miR-520g mediated the resistance of colorectal cancer cells to 5-fluorouracil (5-FU) or oxaliplatin by downregulating CDKN1A expression [58]. These studies suggest that the presence of CDKN1A protects cancer cells from apoptosis after anti-cancer therapy. Therefore, in this study, the changes in CDKN1A expression were caused by chemical drugs, which may stimulate the translocation of CDKN1A protein from the nucleus to the cytoplasm, thereby activating downstream-related pathways to reduce the sensitivity of cells to chemotherapy drugs; however, the specific mechanism is still unknown. In short, the mechanisms of interaction between cell signalling pathways and epigenetic elements are diverse and complex and merit further exploration and verification.

This study still has some shortcomings, such as the small sample size and lack of follow-up data. The direct intermolecular regulatory mechanism by which METTL3 affects tumour progression and survival was not clarified in detail, and we will be further studied in the future.

## Conclusion

Our data suggest the importance of chemotherapy-induced alterations in m^6^A modifications and the protective role of METTL3 in HR+HER2− subtype BC, providing a theoretical basis for METTL3 as a new predictor of chemotherapy response and target for drug therapy.

## Supplementary Information

**Additional file 1.** METTL3 is decreased in HR+HER2− BC samples and cell lines. **A** The half-maximal inhibitory concentration (IC50) values of DOX, PTX and Cis for MCF-7 and T47D cells, respectively. **B** The expression of m6A-related genes in the GSE763 cohort. **C** The protein levels of METTL3 in paired HR+/HER2− BC tissues obtained before and after chemotherapy treatment. **D** and **E** The mRNA and protein levels of METTL3 in MCF-7 and T47D cells treated with different concentrations PTX and Cis for 24 h and their corresponding control cells.

**Additional file 2.** METTL3 reduction enhances T47D cells growth. **A** m6A levels in T47D cells treated with DOX, PTX or Cis for 24 h compared with those in the control cells. **B** m6A levels in MDA-MB-231 cells treated with DOX for 24 h compared with those in the control cells. **C** The protein and mRNA levels of METTL3 in T47D cells with knockdown or overexpression of METTL3 were measured by western blotting and qRT-PCR, respectively. **D** Knockdown of METTL3 improved the proliferation ability of T47D cells in the presence or absence of DOX for 24 h. **E** Overexpression of METTL3 impaired the proliferation ability of T47D cells in the presence or absence of DOX for 24 h. **F** Knockdown of METTL3 improved the colony formation ability of T47D cells in the presence or absence of DOX for 24 h (left panel). Quantification of the colony formation assay results (right panel). **G** Overexpression of METTL3 impaired the colony formation ability of T47D cells in the presence or absence of DOX for 24 h (left panel). Quantification of the colony formation assay results (right panel). **H** and **I** Cell cycle distribution of T47D cells with knockdown or overexpression of METTL3 was analysed by flow cytometry. Ap, apoptosis phase; G1, DNA pre-synthesis phase; S, DNA synthesis phase; G2, DNA post-synthesis phase.

**Additional file 3.** Depletion of METTL3 drives T47D cells migration and invasion in vitro. **A** Knockdown or overexpression of METTL3 affects T47D cell migration in vitro as indicated by the wound healing assay. **B** Knockdown or overexpression of METTL3 affects T47D cell migration and invasion in vitro as indicated by the Transwell assay. **C** Overexpression of METTL3 promotes T47D cell apoptosis.

**Additional file 4.** CDKN1A is a potential downstream target and HNRNPA2B1 is a potential reader gene. **A** Bibliometric analysis suggested that CDKN1A is one of the key molecules in the PI3K/AKT pathway. **B** Venn diagram showing only the HNRNPA2B1 gene with differential expression between the GSE763 and GSE87455. **C** The mRNA levels of HNRNPA2B1 in MCF-7 cells treated with different concentrations DOX for 24 h and their corresponding control cells.

## Data Availability

The data sets analysed during the current study are available in the Gene Expression Omnibus repository (https://www.ncbi.nlm.nih.gov/geo/).

## Abbreviations

BC: Breast cancer
HRs: Hormone receptors
HER2: Human epidermal growth factor receptor 2
HR+HER2−: HR-positive and HER2-negative
AIs: Aromatase inhibitors
PFS: Progression-free survival
pCR: Pathological complete response
DFS: Disease-free survival
OS: Overall survival
m^6^A: *N*^6^-Methyladenosine
mRNA: Messenger RNA
METTL3: Methyltransferase-like 3
METTL14: Methyltransferase-like 14
WTAP: Wilms tumour 1-associated protein
FTO: Fat mass and obesity-associated
ALKBH5: AlkB homolog 5
BCSCs: BC stem cells
TNBC: Triple-negative BC
DOX: Doxorubicin
CDKN1A: Cyclin-dependent inhibitor kinase 1A
EMT: Epithelial-mesenchymal transition
shRNA: Short-hairpin RNA
MeRIP: Methylated RNA immunoprecipitation
GO: Gene Ontology
KEGG: Kyoto Encyclopedia of Genes and Genomes
DEGs: Differentially expressed genes
PTX: Paclitaxel
Cis: Cisplatin

## References

[1] Giuliano M, Trivedi MV, Schiff R (2013). Bidirectional crosstalk between the estrogen receptor and human epidermal growth factor receptor 2 signaling pathways in breast cancer: molecular basis and clinical implications. Breast Care (Basel, Switzerland).

[2] Ouyang D, Su J, Huang P, Li M, Li Q, Zhao P, Chen Q, Zou Q, Feng X, Qian K (2018). Identification of lncRNAs via microarray analysis for predicting HER2-negative breast cancer response to neoadjuvant chemotherapy. Int J Clin Exp Pathol.

[3] Harbeck N, Penault-Llorca F, Cortes J, Gnant M, Houssami N, Poortmans P, Ruddy K, Tsang J, Cardoso F (2019). Breast cancer. Nat Rev Dis Primers.

[4] Gradishar WJ, Moran MS, Abraham J, Aft R, Agnese D, Allison KH, Blair SL, Burstein HJ, Dang C, Elias AD (2021). NCCN Guidelines® insights: breast cancer, version 4.2021. J Natl Compr Cancer Netw JNCCN.

[5] Aromatase inhibitors versus tamoxifen in early breast cancer: patient-level meta-analysis of the randomised trials. Lancet (London, England). 2015;386(10001):1341–52. 10.1016/S0140-6736(15)61074-1 26211827

[6] Mamounas EP, Tang G, Paik S, Baehner FL, Liu Q, Jeong JH, Kim SR, Butler SM, Jamshidian F, Cherbavaz DB (2018). 21-Gene recurrence score for prognosis and prediction of taxane benefit after adjuvant chemotherapy plus endocrine therapy: results from NSABP B-28/NRG oncology. Breast Cancer Res Treat.

[7] Gradishar WJ, Anderson BO, Abraham J, Aft R, Agnese D, Allison KH, Blair SL, Burstein HJ, Dang C, Elias AD (2020). Breast cancer, version 3.2020, NCCN clinical practice guidelines in oncology. J Natl Compr Cancer Netw JNCCN.

[8] Park IH, Lee KS, Ro J (2015). Effects of second and subsequent lines of chemotherapy for metastatic breast cancer. Clin Breast Cancer.

[9] Dey N, Aske J, De P (2021). Targeted neoadjuvant therapies in HR+/HER2− breast cancers: challenges for improving pCR. Cancers.

[10] He L, Li H, Wu A, Peng Y, Shu G, Yin G (2019). Functions of N6-methyladenosine and its role in cancer. Mol Cancer.

[11] Wang X, Feng J, Xue Y, Guan Z, Zhang D, Liu Z, Gong Z, Wang Q, Huang J, Tang C (2016). Structural basis of N(6)-adenosine methylation by the METTL3-METTL14 complex. Nature.

[12] Yang Y, Hsu PJ, Chen YS, Yang YG (2018). Dynamic transcriptomic m(6)A decoration: writers, erasers, readers and functions in RNA metabolism. Cell Res.

[13] Zhao Y, Shi Y, Shen H, Xie W (2020). m(6)A-binding proteins: the emerging crucial performers in epigenetics. J Hematol Oncol.

[14] Chen XY, Zhang J, Zhu JS (2019). The role of m(6)A RNA methylation in human cancer. Mol Cancer.

[15] Sun T, Wu Z, Wang X, Wang Y, Hu X, Qin W, Lu S, Xu D, Wu Y, Chen Q (2020). LNC942 promoting METTL14-mediated m(6)A methylation in breast cancer cell proliferation and progression. Oncogene.

[16] Chang G, Shi L, Ye Y, Shi H, Zeng L, Tiwary S, Huse JT, Huo L, Ma L, Ma Y (2020). YTHDF3 induces the translation of m(6)A-enriched gene transcripts to promote breast cancer brain metastasis. Cancer Cell.

[17] Wang S, Zou X, Chen Y, Cho WC, Zhou X (2020). Effect of N6-methyladenosine regulators on progression and prognosis of triple-negative breast cancer. Front Genet.

[18] Petri BJ, Piell KM, South Whitt GC, Wilt AE, Poulton CC, Lehman NL, Clem BF, Nystoriak MA, Wysoczynski M, Klinge CM (2021). HNRNPA2B1 regulates tamoxifen- and fulvestrant-sensitivity and hallmarks of endocrine resistance in breast cancer cells. Cancer Lett.

[19] Shi Y, Zheng C, Jin Y, Bao B, Wang D, Hou K, Feng J, Tang S, Qu X, Liu Y (2020). Reduced expression of METTL3 promotes metastasis of triple-negative breast cancer by m6A methylation-mediated COL3A1 up-regulation. Front Oncol.

[20] Zhang C, Samanta D, Lu H, Bullen JW, Zhang H, Chen I, He X, Semenza GL (2016). Hypoxia induces the breast cancer stem cell phenotype by HIF-dependent and ALKBH5-mediated m^6^A-demethylation of NANOG mRNA. Proc Natl Acad Sci USA.

[21] Zhang C, Zhi WI, Lu H, Samanta D, Chen I, Gabrielson E, Semenza GL (2016). Hypoxia-inducible factors regulate pluripotency factor expression by ZNF217- and ALKBH5-mediated modulation of RNA methylation in breast cancer cells. Oncotarget.

[22] Klinge CM, Piell KM, Tooley CS, Rouchka EC (2019). HNRNPA2/B1 is upregulated in endocrine-resistant LCC9 breast cancer cells and alters the miRNA transcriptome when overexpressed in MCF-7 cells. Sci Rep.

[23] Liu X, Gonzalez G, Dai X, Miao W, Yuan J, Huang M, Bade D, Li L, Sun Y, Wang Y (2020). Adenylate kinase 4 modulates the resistance of breast cancer cells to tamoxifen through an m(6)A-based epitranscriptomic mechanism. Mol Ther J Am Soc Gene Ther.

[24] Samanta S, Pursell B, Mercurio AM (2013). IMP3 protein promotes chemoresistance in breast cancer cells by regulating breast cancer resistance protein (ABCG2) expression. J Biol Chem.

[25] Loi S, Drubay D, Adams S, Pruneri G, Francis PA, Lacroix-Triki M, Joensuu H, Dieci MV, Badve S, Demaria S (2019). Tumor-infiltrating lymphocytes and prognosis: a pooled individual patient analysis of early-stage triple-negative breast cancers. J Clin Oncol Off J Am Soc Clin Oncol.

[26] Yang X, Shang P, Yu B, Jin Q, Liao J, Wang L, Ji J, Guo X (2021). Combination therapy with miR34a and doxorubicin synergistically inhibits Dox-resistant breast cancer progression via down-regulation of Snail through suppressing Notch/NF-κB and RAS/RAF/MEK/ERK signaling pathway. Acta Pharm Sin B.

[27] Krakhmal NV, Zavyalova MV, Denisov EV, Vtorushin SV, Perelmuter VM (2015). Cancer invasion: patterns and mechanisms. Acta Nat.

[28] Kashiwagi S, Yashiro M, Takashima T, Aomatsu N, Ikeda K, Ogawa Y, Ishikawa T, Hirakawa K (2011). Advantages of adjuvant chemotherapy for patients with triple-negative breast cancer at Stage II: usefulness of prognostic markers E-cadherin and Ki67. Breast Cancer Res BCR.

[29] Brandão M, Caparica R, Eiger D, de Azambuja E (2019). Biomarkers of response and resistance to PI3K inhibitors in estrogen receptor-positive breast cancer patients and combination therapies involving PI3K inhibitors. Ann Oncol Off J Eur Soc Med Oncol.

[30] Bodai BI, Tuso P (2015). Breast cancer survivorship: a comprehensive review of long-term medical issues and lifestyle recommendations. Perm J.

[31] Asaoka M, Gandhi S, Ishikawa T, Takabe K (2020). Neoadjuvant chemotherapy for breast cancer: past, present, and future. Breast Cancer Basic Clin Res.

[32] Müller C, Schmidt G, Juhasz-Böss I, Jung L, Huwer S, Solomayer EF, Juhasz-Böss S (2021). Influences on pathologic complete response in breast cancer patients after neoadjuvant chemotherapy. Arch Gynecol Obstet.

[33] Han J, Wang JZ, Yang X, Yu H, Zhou R, Lu HC, Yuan WB, Lu JC, Zhou ZJ, Lu Q (2019). METTL3 promote tumor proliferation of bladder cancer by accelerating pri-miR221/222 maturation in m6A-dependent manner. Mol Cancer.

[34] Li T, Hu PS, Zuo Z, Lin JF, Li X, Wu QN, Chen ZH, Zeng ZL, Wang F, Zheng J (2019). METTL3 facilitates tumor progression via an m(6)A-IGF2BP2-dependent mechanism in colorectal carcinoma. Mol Cancer.

[35] Li HB, Tong J, Zhu S, Batista PJ, Duffy EE, Zhao J, Bailis W, Cao G, Kroehling L, Chen Y (2017). m(6)A mRNA methylation controls T cell homeostasis by targeting the IL-7/STAT5/SOCS pathways. Nature.

[36] Jin D, Guo J, Wu Y, Du J, Yang L, Wang X, Di W, Hu B, An J, Kong L (2019). m(6)A mRNA methylation initiated by METTL3 directly promotes YAP translation and increases YAP activity by regulating the MALAT1-miR-1914-3p-YAP axis to induce NSCLC drug resistance and metastasis. J Hematol Oncol.

[37] Hao L, Wang JM, Liu BQ, Yan J, Li C, Jiang JY, Zhao FY, Qiao HY, Wang HQ (2021). m6A-YTHDF1-mediated TRIM29 upregulation facilitates the stem cell-like phenotype of cisplatin-resistant ovarian cancer cells. Biochim Biophys Acta Mol Cell Res.

[38] Deng X, Su R, Weng H, Huang H, Li Z, Chen J (2018). RNA N(6)-methyladenosine modification in cancers: current status and perspectives. Cell Res.

[39] Chen M, Wei L, Law CT, Tsang FH, Shen J, Cheng CL, Tsang LH, Ho DW, Chiu DK, Lee JM (2018). RNA N6-methyladenosine methyltransferase-like 3 promotes liver cancer progression through YTHDF2-dependent posttranscriptional silencing of SOCS2. Hepatology (Baltimore, MD).

[40] Wang X, Lu Z, Gomez A, Hon GC, Yue Y, Han D, Fu Y, Parisien M, Dai Q, Jia G (2014). N6-methyladenosine-dependent regulation of messenger RNA stability. Nature.

[41] Cai X, Wang X, Cao C, Gao Y, Zhang S, Yang Z, Liu Y, Zhang X, Zhang W, Ye L (2018). HBXIP-elevated methyltransferase METTL3 promotes the progression of breast cancer via inhibiting tumor suppressor let-7g. Cancer Lett.

[42] Cui Q, Shi H, Ye P, Li L, Qu Q, Sun G, Sun G, Lu Z, Huang Y, Yang CG (2017). m(6)A RNA methylation regulates the self-renewal and tumorigenesis of glioblastoma stem cells. Cell Rep.

[43] Vu LP, Pickering BF, Cheng Y, Zaccara S, Nguyen D, Minuesa G, Chou T, Chow A, Saletore Y, MacKay M (2017). The N(6)-methyladenosine (m(6)A)-forming enzyme METTL3 controls myeloid differentiation of normal hematopoietic and leukemia cells. Nat Med.

[44] Visvanathan A, Patil V, Arora A, Hegde AS, Arivazhagan A, Santosh V, Somasundaram K (2018). Essential role of METTL3-mediated m(6)A modification in glioma stem-like cells maintenance and radioresistance. Oncogene.

[45] Wang H, Xu B, Shi J (2020). N6-methyladenosine METTL3 promotes the breast cancer progression via targeting Bcl-2. Gene.

[46] Xie J, Ba J, Zhang M, Wan Y, Jin Z, Yao Y (2021). The m6A methyltransferase METTL3 promotes the stemness and malignant progression of breast cancer by mediating m6A modification on SOX2. J BUON Off J Balkan Union Oncol.

[47] Pan X, Hong X, Li S, Meng P, Xiao F (2021). METTL3 promotes adriamycin resistance in MCF-7 breast cancer cells by accelerating pri-microRNA-221-3p maturation in a m6A-dependent manner. Exp Mol Med.

[48] Li S, Jiang F, Chen F, Deng Y, Pan X (2022). Effect of m6A methyltransferase METTL3-mediated MALAT1/E2F1/AGR2 axis on adriamycin resistance in breast cancer. J Biochem Mol Toxicol.

[49] Patil DP, Pickering BF, Jaffrey SR (2018). Reading m(6)A in the transcriptome: m(6)A-binding proteins. Trends Cell Biol.

[50] Liu Y, Li H, Liu F, Gao LB, Han R, Chen C, Ding X, Li S, Lu K, Yang L (2020). Heterogeneous nuclear ribonucleoprotein A2/B1 is a negative regulator of human breast cancer metastasis by maintaining the balance of multiple genes and pathways. EBioMedicine.

[51] Alarcón CR, Goodarzi H, Lee H, Liu X, Tavazoie S, Tavazoie SF (2015). HNRNPA2B1 Is a mediator of m(6)A-dependent nuclear RNA processing events. Cell.

[52] Xu X, Lai Y, Hua ZC (2019). Biosci Rep.

[53] Harper JW, Adami GR, Wei N, Keyomarsi K, Elledge SJ (1993). The p21 Cdk-interacting protein Cip1 is a potent inhibitor of G1 cyclin-dependent kinases. Cell.

[54] Stivala LA, Cazzalini O, Prosperi E (2012). The cyclin-dependent kinase inhibitor p21CDKN1A as a target of anti-cancer drugs. Curr Cancer Drug Targets.

[55] Abella N, Brun S, Calvo M, Tapia O, Weber JD, Berciano MT, Lafarga M, Bachs O, Agell N (2010). Nucleolar disruption ensures nuclear accumulation of p21 upon DNA damage. Traffic (Copenhagen, Denmark).

[56] Abbas T, Dutta A (2009). p21 in cancer: intricate networks and multiple activities. Nat Rev Cancer.

[57] Xu S, Huang H, Chen YN, Deng YT, Zhang B, Xiong XD, Yuan Y, Zhu Y, Huang H, Xie L (2016). DNA damage responsive miR-33b-3p promoted lung cancer cells survival and cisplatin resistance by targeting p21(WAF1/CIP1). Cell Cycle (Georgetown, Tex).

[58] Zhang Y, Geng L, Talmon G, Wang J (2015). MicroRNA-520g confers drug resistance by regulating p21 expression in colorectal cancer. J Biol Chem.

