# Supplementary figures and images for: METTL3 depletion contributes to tumour progression and drug resistance via N6 methyladenosine-dependent mechanism in HR+HER2—breast cancer
Source: Breast Cancer Res. 2023 Feb 10;25:19. doi: 10.1186/s13058-022-01598-w (PMC9921123; doi:10.1186/s13058-022-01598-w)

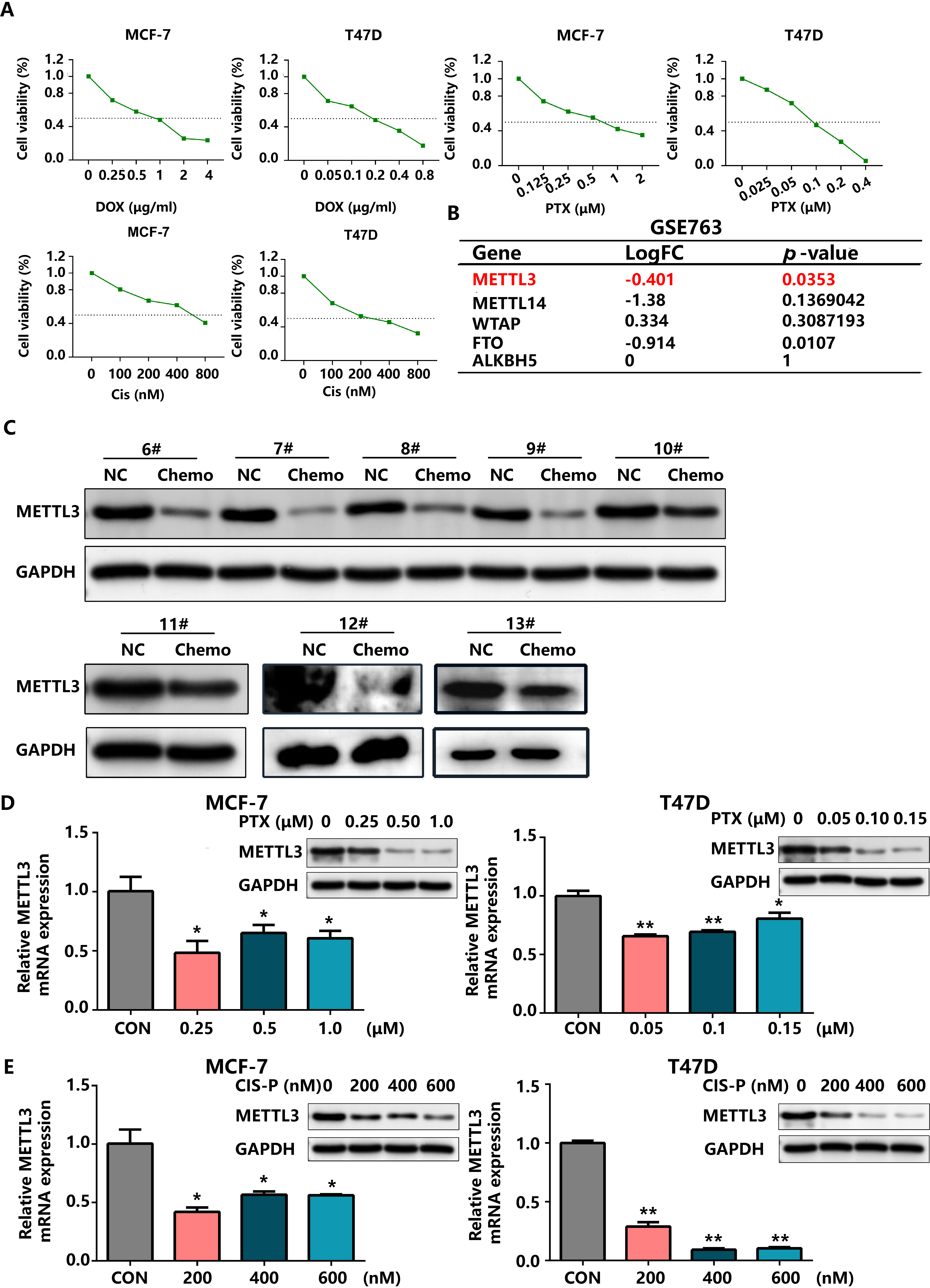

Supplement: Supplementary file 1 — Additional file 1. METTL3 is decreased in HR+HER2− BC samples and cell lines. A The half-maximal inhibitory concentration (IC50) values of DOX, PTX and Cis for MCF-7 and T47D cells, respectively. B The expression of m6A-related genes in the GSE763 cohort. C The protein levels of METTL3 in paired HR+/HER2− BC tissues obtained before and after chemotherapy treatment. D and E The mRNA and protein levels of METTL3 in MCF-7 and T47D cells treated with different concentrations PTX and Cis for 24 h and their corresponding control cells. [file 13058_2022_1598_MOESM1_ESM.tif]

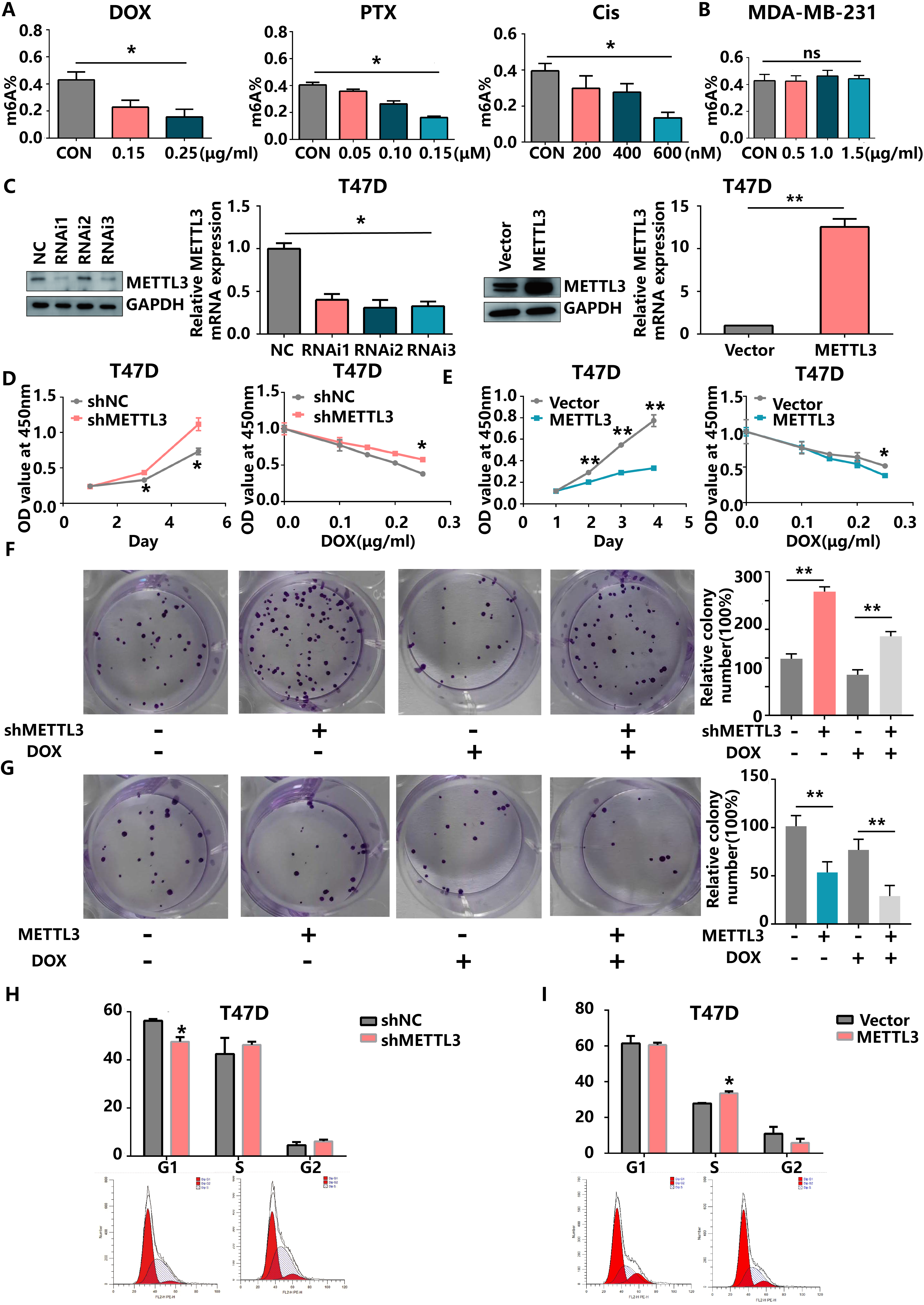

Supplement: Supplementary file 2 — Additional file 2. METTL3 reduction enhances T47D cells growth. A m6A levels in T47D cells treated with DOX, PTX or Cis for 24 h compared with those in the control cells. B m6A levels in MDA-MB-231 cells treated with DOX for 24 h compared with those in the control cells. C The protein and mRNA levels of METTL3 in T47D cells with knockdown or overexpression of METTL3 were measured by western blotting and qRT-PCR, respectively. D Knockdown of METTL3 improved the proliferation ability of T47D cells in the presence or absence of DOX for 24 h. E Overexpression of METTL3 impaired the proliferation ability of T47D cells in the presence or absence of DOX for 24 h. F Knockdown of METTL3 improved the colony formation ability of T47D cells in the presence or absence of DOX for 24 h (left panel). Quantification of the colony formation assay results (right panel). G Overexpression of METTL3 impaired the colony formation ability of T47D cells in the presence or absence of DOX for 24 h (left panel). Quantification of the colony formation assay results (right panel). H and I Cell cycle distribution of T47D cells with knockdown or overexpression of METTL3 was analysed by flow cytometry. Ap, apoptosis phase; G1, DNA pre-synthesis phase; S, DNA synthesis phase; G2, DNA post-synthesis phase. [file 13058_2022_1598_MOESM2_ESM.tif]

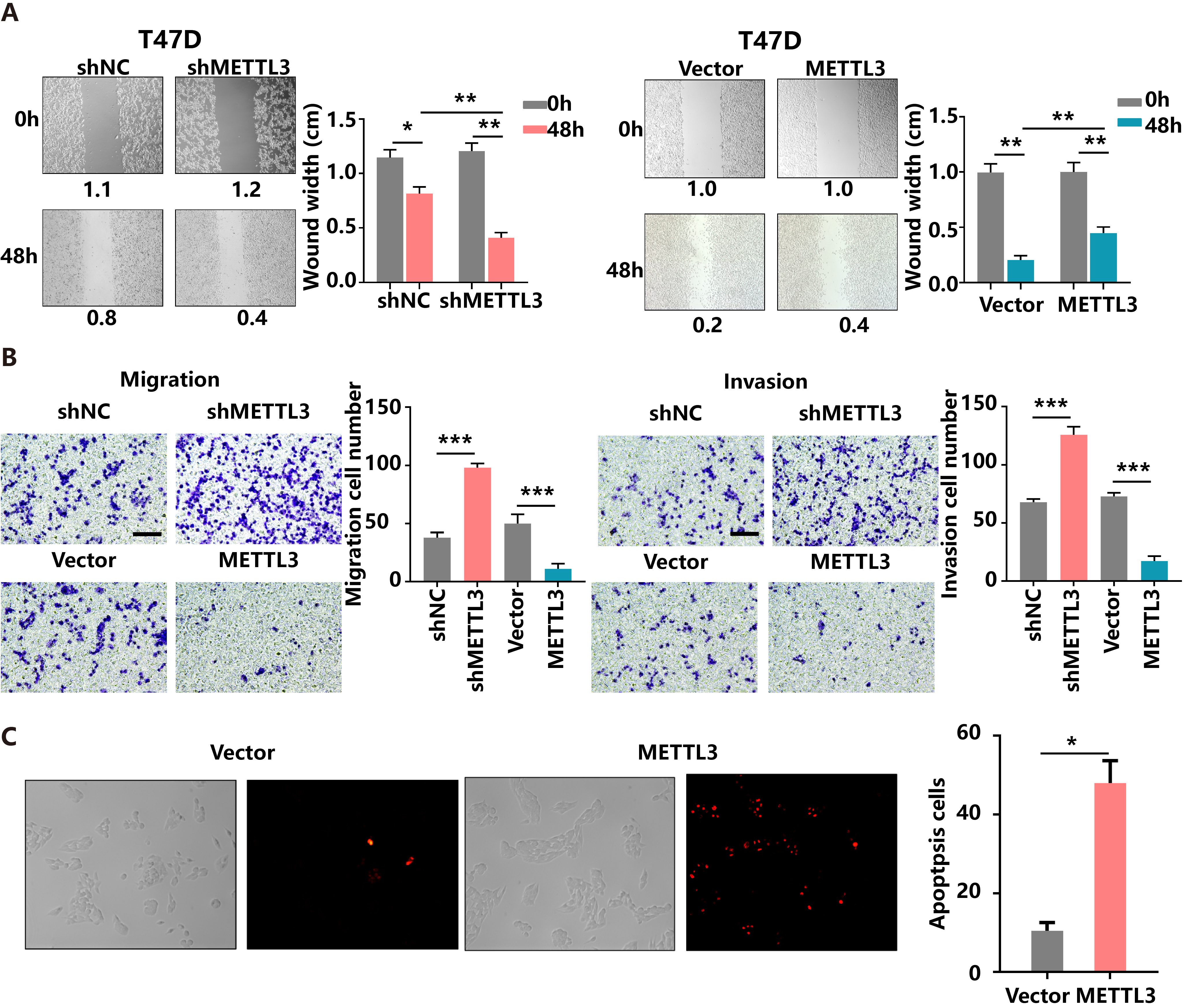

Supplement: Supplementary file 3 — Additional file 3. Depletion of METTL3 drives T47D cells migration and invasion in vitro. A Knockdown or overexpression of METTL3 affects T47D cell migration in vitro as indicated by the wound healing assay. B Knockdown or overexpression of METTL3 affects T47D cell migration and invasion in vitro as indicated by the Transwell assay. C Overexpression of METTL3 promotes T47D cell apoptosis. [file 13058_2022_1598_MOESM3_ESM.tif]

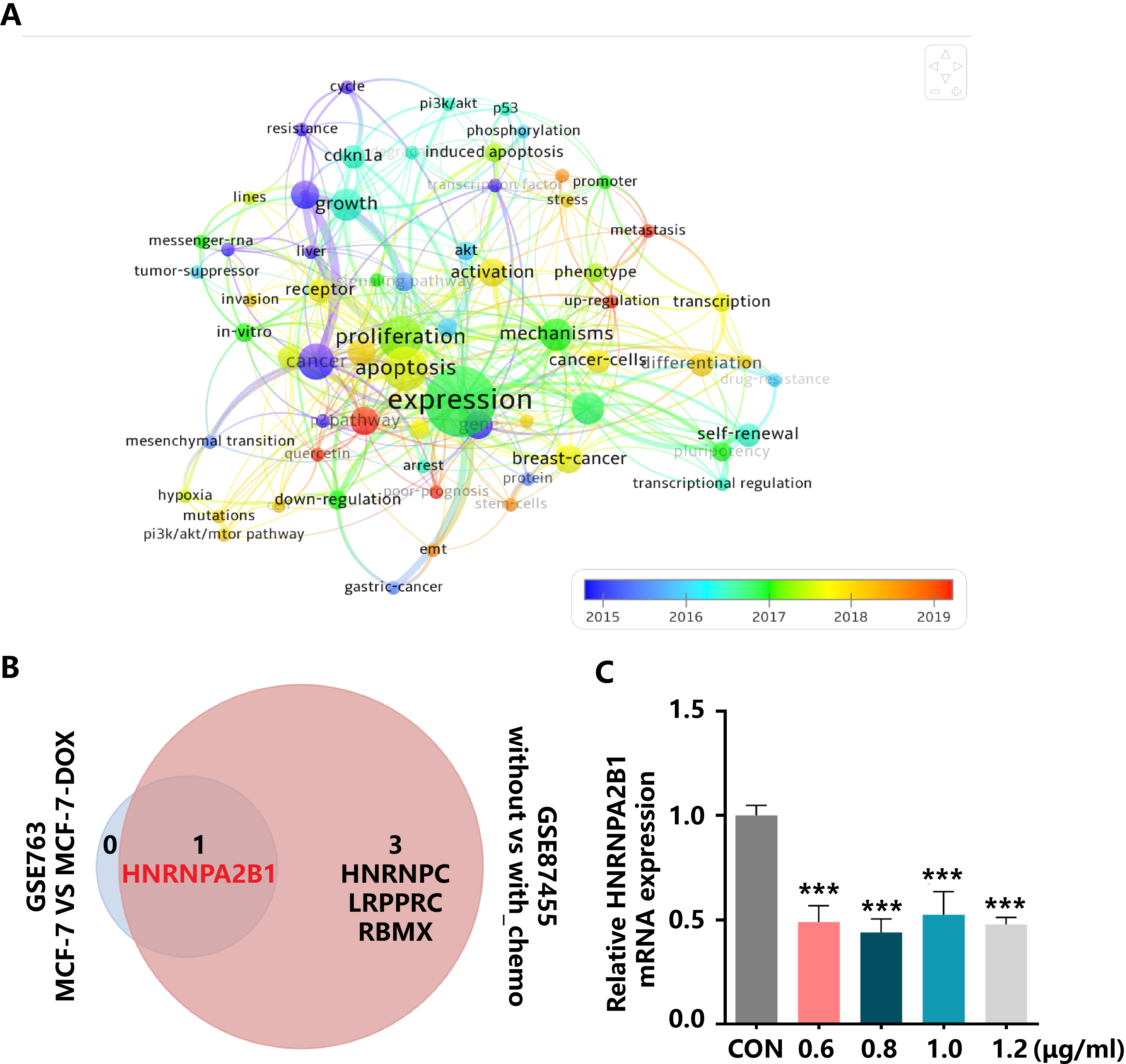

Supplement: Supplementary file 4 — Additional file 4. CDKN1A is a potential downstream target and HNRNPA2B1 is a potential reader gene. A Bibliometric analysis suggested that CDKN1A is one of the key molecules in the PI3K/AKT pathway. B Venn diagram showing only the HNRNPA2B1 gene with differential expression between the GSE763 and GSE87455. C The mRNA levels of HNRNPA2B1 in MCF-7 cells treated with different concentrations DOX for 24 h and their corresponding control cells. [file 13058_2022_1598_MOESM4_ESM.tif]
